# P53 Triple antiphospholipid antibody positive renal thrombotic microangiopathy in a patient with proliferative lupus nephritis - a case report

**DOI:** 10.1093/rap/rkae117.084

**Published:** 2024-11-05

**Authors:** Ranitha Gopi, Weena Stanley, Shashikala Surkunda

**Affiliations:** Kasturba Medical College, Manipal Academy of Higher Education, Manipal, India; Basildon University Hospital, Mid and South Essex NHS Trust, Basildon, United Kingdom; Kasturba Medical College, Manipal Academy of Higher Education, Manipal, India; Kasturba Medical College, Manipal Academy of Higher Education, Manipal, India

## Abstract

**Introduction:**

The presence of renal microvascular lesions in lupus nephritis is well recognized as a major prognostic factor which can also determine the choice of treatment. Prevalence of renal microvascular lesions in lupus nephritis can range from 53.4 to 81.8% and of the five described types of renal vasculopathy, renal thrombotic microangiopathy (TMA) is an important microvascular lesion. Antiphospholipid antibodies (aPL) are a well-recognized etiology for the same. The presence of triple antiphospholipid antibody positive TMA in lupus nephritis is a rare association and we will be describing the clinical course and management of a patient who presented with the same.

**Case description:**

A 36-year-old South-Indian female presented to our Outpatient Department with a history of multiple joint pains for 3 months and intermittent episodes of fever following the onset of joint pains. She had no other positive history and no significant past medical history. She had no previous history of abortions and had two uneventful pregnancies in the past. After history and examination, she was diagnosed to have inflammatory polyarthritis. She was also noted to have pitting pedal oedema.

Initial blood investigations revealed microcytic hypochromic anaemia of 91 g/L with normal leucocyte and platelet counts. Her renal and liver function tests were within normal limits. However, she was noted to have a mild hypoalbuminemia of 32 g/L. Complement levels were reduced.

A 24-hour urine protein showed proteinuria of 588 mg/24 hours. Antinuclear antibody testing was strongly positive and an ENA screen was positive for antibodies against SS-A, SS-B and Ro-52. The patient was diagnosed to have systemic lupus erythematosus (SLE) and was started on hydroxychloroquine and oral prednisolone at 0.5 mg/kg.

Kidney biopsy was suggestive of diffuse proliferative lupus nephritis with evidence of thrombotic microangiopathy along with near total luminal occlusion of arterioles and crescent formation (<50%). Antiphospholipid antibody profile was positive for anticardiolipin antibodies IgG and IgM, anti-beta-2-glycoprotein-I (anti-β2GPI) antibodies and lupus anticoagulant. The patient received intravenous methylprednisolone pulse therapy at 15 mg/kg/24 hours for three days followed by oral prednisolone at 0.5 mg/kg which was planned to be tapered by 2.5 mg every two weeks after one month. She was initiated on mycophenolate mofetil at 1 gram twice daily along with warfarin which was planned to be continued for 12 months. Following this, we planned to switch to aspirin if no thrombotic manifestations had occurred. Her kidney function after 8 weeks was stable with overall symptomatic improvement.

**Discussion:**

Lupus nephritis with renal thrombotic microangiopathy has been found to have worse outcomes in terms of mortality and remission rates. Studies on lupus nephritis-combined TMA pointed towards serum anti-phospholipid antibodies as a major etiologic factor. Antiphospholipid antibodies (aPL) are a group of antibodies against phospholipids or phospholipid-binding proteins, which includes anticardiolipin (aCL), anti-b2-glycoprotein I (anti-b2GPI) antibodies as well as lupus anticoagulant. To make a diagnosis of antiphospholipid syndrome (APS) nephropathy, one of the following needs to be present on the biopsy: thrombotic microangiopathy, interlobular fibrous intimal hyperplasia, arterial and arteriolar recanalizing thrombi, fibrous arterial occlusion and and focal cortical atrophy. TMA is an acute and severe manifestation of APS nephropathy.

Lupus anticoagulant is the antibody most associated with APS nephropathy but anticardiolipin antibodies (aCL) have also been demonstrated to have a close association.Incidence of renal involvement is thought to be higher in patients with double or triple antibody positivity but there is limited data on antibody profile and its prognostic implications. However, presence of lupus anticoagulant and aCL antibodies have been associated with intraglomerular microthrombi formation in lupus nephritis with TMA, which is a poor prognostic factor.

There is contradictory results on whether the presence of aPL antibodies in lupus nephritis with renal vasculopathy can significantly influence prognosis. In a case series of 33 patients with diffuse lupus nephritis and thrombotic microangiopathy in China, Hu et al found that there was no significant difference in outcomes between aPL positive versus aPL negative patients. However, another study found that aPL positive patients had worse outcomes in terms of remission and mortality. In their review, Azevedo et al advocates aggressive intervention in TMA secondary to aPL because of its poor prognosis.

Our patient was treated with standard immunosuppression for proliferative lupus nephritis and only anticoagulation was started for the associated aPL-thrombotic microangiopathy.

**Key learning points:**

• Lupus nephritis with combined TMA should be considered if a patient with lupus develops acute renal injury.

• APS nephropathy is more common in aPL positive than aPL-negative SLE patients.

• Pathophysiology behind aPL induced TMA appears to be mainly complement activation. Studies have demonstrated that inhibition of complement membrane-attack complex can reduce aPL-induced TMA lesions-Eculizumab has been trialled. Clinical manifestations are systemic hypertension, proteinuria and kidney injury.

• Presence of aCL and lupus anticoagulant antibodies are commonly described, however prevalence estimates vary widely. Anti-b2GPI has been infrequently reported, however in a study looking at domain profiling of Anti-b2GPI in relation to APS nephropathy, Sciascia et al found that anti-β2GPI antibody positivity increased risk of developing TMA in patients with concomitant lupus nephritis. Another study by Sciascia et al which included 123 patients with APS nephropathy found that patients with triple aPL positivity had the worst renal prognosis and this same cluster was associated with thrombotic microangiopathy and intra glomerular thrombosis.

• Prognosis of lupus nephritis with combined renal TMA is poor. An Indian study by Pattanashetti et al found that patients with TMA had higher serum creatinine, worse chronic tubulo-interstitial parameters and higher treatment failure rates compared to those without TMA. Prognosis of lupus nephritis with aPL associated TMA is worse and and one reason for that is the higher prevalence of glomerular thrombosis which is associated with aCL antibodies and lupus anticoagulant.

• Anticoagulation is currently the treatment for patients with APS-induced renal lesions. However patients with aPL induced TMA with lupus nephritis are likely to have higher rates of progression to chronic kidney disease and mortality and hence aggressive treatment is warranted. One study by Li et al in a cohort of 70 patients found that plasmapheresis improved long-term outcomes in patients with lupus nephritis combined TMA.

